# Acute kidney injury induced lithium toxicity with concomitant neuroleptic malignant syndrome

**DOI:** 10.37796/2211-8039.1459

**Published:** 2024-09-01

**Authors:** Yin Ye Lai, Normaizuwana Mohamed Mokhtar, Intan Nureslyna Samsudin, Subashini C. Thambiah

**Affiliations:** a Department of Pathology, Faculty of Medicine & Health Sciences, Universiti Putra Malaysia, Malaysia; b Department of Pathology, Hospital Kuala Lumpur, Ministry of Health, Malaysia

**Keywords:** Bipolar disorder, Kidney and urinary tract diseases, Lithium toxicity, NMS

## Abstract

Lithium, despite being an indispensable agent in the treatment of psychiatric disorders, has a narrow therapeutic index and needs to be carefully administered. Neuroleptic malignant syndrome (NMS) is a rare but potentially fatal complication due to central dopaminergic blockade. This case report illustrates the challenges in lithium therapy particularly related to the development of NMS when further risk factors such as polypharmacy and dehydration are present. We report a case of a 50-year-old man with underlying bipolar affective disorder who was previously able to tolerate olanzapine and lithium well, however developed chronic lithium toxicity due to diminished lithium elimination in acute kidney injury following a two-week history of viral acute gastroenteritis. He also developed NMS which could either be triggered independently by olanzapine; lithium toxicity; or attributed by a synergistic combination from lithium and olanzapine which led to an enhanced neurotoxicity in an already unstable dopaminergic pathway. Fluid therapy and supportive care allowed the patient to recover, and he was discharged well with a lower potency neuroleptic with slow dose titration.

## 1. Introduction

Lithium is an inexpensive and widely used mood stabilizer [1] for treatment and prophylaxis of bipolar disorder [1]. In spite of its proven benefits, long-term therapy with lithium is often complicated by a narrow therapeutic index and an elevated risk of toxicity [2]. There are three types of lithium toxicity, namely acute, acute on chronic, and chronic [2]. Acute lithium toxicity is seen when a lithium-naïve person takes an overdose; acute-on-chronic lithium toxicity happens when lithium treated individuals take an overdose; whilst chronic toxicity arises when lithium consumption exceeds excretion on a chronic basis such as when lithium dosage has been escalated or when lithium elimination is diminished in renal impairment [2]. Low neurotoxicity risk is seen with acute lithium toxicity because the rate of distribution into intracellular compartment is low when compared to the excretion rate [2]. This is evidenced by the fact that lithium follows a 2-compartment model with a low initial volume of distribution at 0.3–0.4 L/kg, increasing to 0.7–1L/kg over the next 6–10 h [3]. Acute-on-chronic toxicity confers a neurotoxic risk greater than acute toxicity since some lithium has been dispersed into the intracellular compartment preceding overdose [2], while chronic lithium toxicity carries the highest risk of neurotoxicity because the prolonged time course (usually weeks) heightens delivery of lithium into the central nervous system [2].

NMS is a rare but potentially fatal reaction [4]. Despite generally being related to consumption of high potency conventional antipsychotics in a susceptible host, it can develop in patients taking atypical antipsychotics (second-generation antipsychotics) alone, or a combination of atypical antipsychotics and lithium [4]. NMS is a neuroleptic-induced trigger of neurometabolic dysregulation manifested as increased skeletal muscle contraction, dysfunctional heat dissipation, and hyperactivity of the sympathoadrenal system, leading to a fulminant hypermetabolic syndrome [5].

This case report illustrates the challenges in lithium therapy particularly related to the development of chronic lithium toxicity and NMS when further risk factors such as polypharmacy and dehydration are present.

## 2. Case report

A 50-year-old man, resident of a local nursing home with underlying bipolar affective disorder and Parkinson’s disease, was taken to the Emergency Department with fever, altered mental status and generalized rigidity. Upon further questioning, a 2-week history of diarrhea and poor oral intake was also noted. Nursing home staff claimed that he was compliant to his routine prescriptions of tablet lithium, olanzapine and trihexyphenidyl for his psychiatric disorder which were all started more than a year ago, and there was no recent change in dosage. On admission, he was drowsy, excessively sweating, febrile with a temperature of 39 °C, tachycardic, tachypneic and had a blood pressure of 105/62 mmHg. His pupils were equal and reactive. There was no neck stiffness however generalized rigidity were evident on neurological examination. He appeared dehydrated with dry lips and buccal mucosa. Physical examinations of the chest and abdomen were otherwise unremarkable. An electrocardiogram revealed no acute ischemic changes whereas urgent computed tomography (CT) brain showed age-related mild cerebral atrophy with no evidence of acute intracranial bleed or other significant abnormalities.

Admission bloods were compared with a baseline result six-months ago. Leukocytosis (white cell count 18.2 × 10^9^/L) was evident, however cerebrospinal fluid (CSF) biochemistry were normal and CSF culture was negative (Table 1). Impaired renal function was evidenced by hypernatremia, hyperkalemia, elevated urea and creatinine (Table 1). Creatine kinase (CK) was elevated <5 times upper limit of normal whilst serum lithium at day three of admission was 2.54 mmol/L (therapeutic level 0.6–1.2 mmol/L) (Table 1). Acute kidney injury (AKI) induced lithium toxicity with concomitant NMS was diagnosed. NMS was considered in this case as he fulfilled the Diagnostic and Statistical Manual of Mental Disorder (DSM-5) criteria-presenting with muscular rigidity, hyperthermia, alteration of mental status, lability of sympathetic nervous system, leukocytosis, and CK elevation after a dopamine antagonist trigger [6]. It is well known that both olanzapine and trihexyphenidyl have significant renal elimination. Even though there is a potential elevation of these drugs in renal impairment, the toxic effects to this patient cannot be determined because their concentration were not measured.

Fluid hydration was commenced, and all antipsychotic medications were withheld. At day nine of admission, the patient was no longer disorientated. His fever and generalized hypertonia resolved. Creatinine clearance improved while CK and serum lithium normalized (Table 1). He was discharged with monotherapy-low dose olanzapine (5 mg once daily) due to its lesser side effects and lower reported rate of NMS [4].

## 3. Discussion

In our patient presenting with fever, muscular rigidity and autonomic instability, traumatic and drug-induced rhabdomyolysis were excluded from the clinical history. Tetanus was unlikely as there were no recent cuts or puncture wounds. Neuroleptic-induced heat stroke was also less likely as it is usually seen in a patient post exposure to high ambient temperature or physical exercise, with absent extrapyramidal signs [5]. Malignant hyperthermia was likewise improbable as there was no recent exposure to anesthetic agents. Lithium intoxication was evident from the history of chronic lithium consumption and elevated serum lithium concentration.

An elevated creatinine >3 times from baseline in this patient was indicative of AKI, whereas an elevated urea: creatinine ratio denoted a pre-renal etiology. Hypernatremia was due to water loss in excess of sodium as seen in diarrhea, whilst hyperkalemia, hypermagnesemia and hyperphosphatemia were caused by decreased excretion via renal tubules in AKI. Viral acute gastroenteritis (AGE) was likely from the 2-week history of diarrhea together with a stool culture and sensitivity which returned negative for bacteria. Raised CK albeit non-specific, is common in the early stage of NMS as it reflects myonecrosis secondary to intense muscle contracture [5]. The other laboratory parameter favoring NMS is leukocytosis. Central nervous system infection was ruled out by virtue of normal CSF biochemistry, culture and sensitivity.

A few mechanisms are postulated to cause NMS in our patient. Firstly, NMS may be triggered independently by olanzapine, an atypical antipsychotic, though typical antipsychotics are usually associated with a greater risk of NMS [7]. Secondly, NMS may be triggered by lithium toxicity. Lithium is renally excreted [2]. Thus, in the face of dehydration and AKI, lithium excretion is impaired. Furthermore, when reaching toxic level, lithium inhibits its own excretion, causing a prolonged half-life and results in toxic accumulation in neural tissues [2]. Although rare, lithium induced NMS has been reported in the literature, postulated to be due to its activity of impeding cyclic adenosine mono-phosphate buildup and consequently altering dopamine activity [3,8]. Thirdly, the patient may have developed additive neurotoxicity from lithium and olanzapine combination leading to NMS whereby the synergistic combination of lithium and neuroleptic drug resulted in an enhanced neurotoxicity in our patient’s already unstable dopaminergic pathway. Either way, all assumptions surround and support the central dopamine receptor blockade theory of NMS.

Risk factors that may contribute to the development of NMS in these individuals include male sex (twice more likely to develop NMS compared to females) [7], underlying age related neurodegenerative or neurological defects [7], dehydration [1,4] and concomitant use of lithium and neuroleptics [3,4], all of which were seen in our patient. Even though risk factors for NMS have been identified, they are mostly non-specific and are relatively common to many disorders. The low incidence of NMS (0.01%–0.02%) [9] makes predictions based on the occurrence of these risk factors difficult. More importantly, risk factors do not function independently or carry equal weight to the development of NMS. Instead, there may be a dynamic interaction amongst them, potentiating each other and changing throughout an individual’s lifespan. This may also explain the fact that although lithium and neuroleptics are commonly prescribed drugs in patients with bipolar disorder, NMS is not as frequently encountered.

The basic principle of lithium intoxication management is enhanced elimination via fluid therapy to reestablish GFR and maximize excretion of lithium [2]. Lithium, due to its low volume of distribution, non-binding to plasma proteins and excellent water solubility, is readily dialyzed [2]. Therefore, in lithium poisoning, when the serum lithium level is > 4.00 mmol/L or, when serious neurological manifestations with an unstable hemodynamic status are observed in a patient with serum lithium level of >2.50 mmol/L, consideration for hemodialysis is pertinent [10]. In the case of our patient, albeit lithium level was raised at 2.54 mmol/L at day 3 of admission, hemodialysis was not done as fluid rehydration had commenced much earlier with substantial improvement in renal profile and urine output. The management of NMS on the other hand is mainly supportive care with removal of the offending drugs and usage of a lower potency neuroleptic with slow titration of the dose [4], as seen in our patient.

## 4. Conclusion

Lithium concentration needs to be maintained within a narrow therapeutic range via careful administration and regular monitoring. Patients on lithium therapy who are suddenly unwell with a decline in glomerular filtration rate (GFR) should be considered the possibility of lithium toxicity. NMS, although rare, may be a fatal syndrome that can arise in patients treated with neuroleptics, lithium or a combination of neuroleptics and lithium. Early diagnosis and treatment are vital to safeguard the well-being of these patients and reduce the mortality of this potentially lethal complication.

## Figures and Tables

**Table 1:** Baseline and current investigation results.

Parameters	Baseline	On admission	Day 3 of admission	Day 5 of admission	Day 9 of admission
Haematology					
Hb (13.0–17.0 g/dL)	–	14.8	–	–	–
WBC (4.0–10.0 × 10^9^/L)	–	18.2	–	–	–
Plt (150–450 × 10^9^/L)	–	310	–	–	–
Renal profile					
Urea (2.8–8.1 mmol/L)	3.8	32.5	16	12.2	9.3
Na (134–146 mmol/L)	147	152	170	160	148
K (3.7–5.0 mmol/L)	3.9	5.1	4.8	4.1	3.7
Cl (98–113 mmol/L)	109	117	141	130	114
CREA (27–87 μmol/L)	105	360	251	175	125
eGFR (ml/min/1.73m^2^)	71	16	25	38	56
Other investigation results					
Ca (2.15–2.50 mmol/L)	2.48	2.33	–	–	–
Mg (0.66–1.07 mmol/L)	1.15	1.65	–	–	–
Phos (0.81–1.45 mmol/L)	1.05	1.64	–	–	–
Glc (<7.8 mmol/L)	–	5.8	–	–	–
CK (<190 U/L)	–	906	599	300	151
Li (0.60–1.20 mmol/L)	–	2.54	1.91	1.47	0.11
CSF for biochemistry					
CSF -appearance Clear	–	–	–		
CSF for -glucose (2.23.9 mmol/L)	5.2	–	–	–	
CSF for protein -(0.15–0.45 g/L)	0.34	–	–	–	
Culture and sensitivity					
CSF for culture - and sensitivity	–	No growth detected	–	–	–
Blood for culture and sensitivity	–	No growth detected	–	–	–
Stool for culture and sensitivity	–	No growth detected	–	–	–
Urine for culture and sensitivity	–	No growth detected	–	–	–

eGFR: estimated glomerular filtration rate based on Chronic Kidney Disease Epidemiology Collaboration.

## References

[1] Patil V Gupta R Verma R Balhara YP Neuroleptic malignant syndrome associated with lithium toxicity Oman Med J 2016 31 309 11 27403245 10.5001/omj.2016.59 PMC4927730

[2] Baird-Gunning J Lea-Henry T Hoegberg LCG Gosselin S Roberts DM Lithium poisoning J Intensive Care Med 2017 32 249 63 27516079 10.1177/0885066616651582

[3] Gill J Singh H Nugent K Acute lithium intoxication and neuroleptic malignant syndrome Pharmacotherapy 2003 23 811 5 12820823 10.1592/phco.23.6.811.32179

[4] Chandran GJ Mikler JR Keegan DL Neuroleptic malignant syndrome: case report and discussion CMAJ (Can Med Assoc J) 2003 169 439 42 12952806 PMC183299

[5] Adnet P Lestavel P Krivosic-Horber R Neuroleptic malignant syndrome Br J Anaesth 2000 85 129 35 10928001 10.1093/bja/85.1.129

[6] American Psychiatric Association Diagnostic and Statistical Manual of Mental Disorders fifth ed 2013 709 11 Arlington volA

[7] Hosseini S Elyasi F Olanzapine-induced neuroleptic malignant syndrome Iran J Med Sci 2017 42 306 9 28533580 PMC5429500

[8] Argyriou AA Drakoulogona O Karanasios P Kouliasa L Leonidou L Giannakopoulou F Lithium-induced fatal neuroleptic malignant syndrome in a patient not being concomitantly treated with commonly offending agents J Pain Symptom Manag 2012 44 e4 6 10.1016/j.jpainsymman.2012.08.010 23217454

[9] Edokpolo O Fyyaz M Lithium toxicity and neurologic effects: probable neuroleptic malignant syndrome resulting from lithium toxicity Case Rep Psychiatry 2012 2012 271858 22953147 10.1155/2012/271858 PMC3420417

[10] Haussmann R Bauer M von Bonin S Grof P Lewitzka U Treatment of lithium intoxication: facing the need for evidence Int J Bipolar Disord 2015 3 23 26493348 10.1186/s40345-015-0040-2 PMC4615994

